# Supplementary material for: The Role of Multiple Marks in Epigenetic Silencing and the Emergence of a Stable Bivalent Chromatin State
Source: PLoS Comput Biol. 2013 Jul 18;9(7):e1003121. doi: 10.1371/journal.pcbi.1003121 (PMC3715441; doi:10.1371/journal.pcbi.1003121)
Supplement: Text S1 — We have relegated the mathematical details of the model in Text S1. Analytical solution of the model in the mean-field limit is presented therein, and the nature of the different phases is summarized. We also compare related models with fewer or more biochemical interactions (parameters) in order to establish that the interactions we have considered are minimal for the engineering design criteria of epigenetic silencing. We also elaborate on Sir perturbations and Sas2 perturbations and discuss the nature of bifurcations/crossovers observed in the phase space. (PDF) [file pcbi.1003121.s001.pdf]

Supplementary Material: The role of multiple marks in epigenetic silencing and the emergence of a stable bivalent chromatin state

Swagatam Mukhopadhyay
Cold Spring Harbor Laboratory, Cold Spring Harbor, NY, USA

Anirvan M. Sengupta
BioMaPS and Department of Physics, Rutgers University, Piscataway, NJ, USA

ANALYTICAL RESULTS

An approximation of the model presented in main text can be solved analytically and is very useful in gaining insight into the nonlinear system and reducing the dimensions of the parameter space. All of the phase diagrams presented in the paper is derived form this analytical solution. We ignore ρ_0 and β_0 and assume that all the decay constants η for the states are equal and rescale time such that the decay constant is unity. At the mean-field level, we replace probabilities by average densities, thereby getting rid of spatial index. Then the set of equations we arrive at are,

$$\frac{dS}{dt} = \rho\, S\,(1 - S - A - M - E) - S, \tag{1}$$
$$\frac{dA}{dt} = \alpha\,(1 - S - A - M - E) - \Gamma\, S\, A - \beta\, E\, A - A, \tag{2}$$
$$\frac{dM}{dt} = \beta E\,(1 - S - A - M - E) - \alpha\, M - M, \tag{3}$$
$$\frac{dE}{dt} = \beta\, E\, A + \alpha\, M - E. \tag{4}$$

We call the model **Model I**. We first discuss the nature of solutions of Model I and point out how they are modified on adding the basal rates ρ_0 and β_0. There are four physically acceptable mean-field solutions, i.e., the probability densities are positive and sum up to unity—

- An *intermediate state* which has high average weight on unmodified and acetylated state and all other states have weight zero. This solution is unstable in most of phase space.

- A *silenced state* which is a silenced state and is one of the bistable states of interest. It has high level of silencing and low level of acetylation marks, but all other marks have weight zero.

- An *active state* which is an active state and the other bistable state of interest. It has high active mark with low levels of methylation and acetylation, and zero level of silencing.

- A *bivalent state* which has both high level of active and silencing mark. This is an unusual state and is stable in some regions of phase space to be described later.

The solutions are

Solution 1: *Intermediate* state

$$S=0, M=0, A=\frac{\alpha}{1+\alpha}, E=0. \tag{5}$$

Solution 2,3: *Active* state and unphysical solution

$$\begin{aligned}
&S=0,\\
&M=\frac{1}{2\sqrt{\alpha}(1+\alpha)\beta}\Big[\mp\sqrt{4\beta+\alpha\left((1+\alpha)^2+2(3+\alpha)\beta+\beta^2\right)}\\
&+\sqrt{\alpha}\left(1+3\beta+\alpha(2+\alpha+\beta)\mp\sqrt{\alpha\left(4\beta+\alpha\left((1+\alpha)^2+2(3+\alpha)\beta+\beta^2\right)\right)}\right)\Big],\\
&A=\frac{2+\alpha(3+\alpha+\beta)\mp\sqrt{\alpha\left(4\beta+\alpha\left((1+\alpha)^2+2(3+\alpha)\beta+\beta^2\right)\right)}}{2(1+\alpha)\beta},\\
&E=\frac{-2+\alpha(-3-\alpha+\beta)\pm\sqrt{\alpha\left(4\beta+\alpha\left((1+\alpha)^2+2(3+\alpha)\beta+\beta^2\right)\right)}}{2(1+\alpha)\beta}.
\end{aligned} \tag{6}$$

Solution 4: *Bivalent* state

$$\begin{aligned}
&S=\frac{(1+\alpha)\beta\left(\alpha\beta+\alpha(1+\alpha+\beta)\rho-(1+\alpha)\rho^2\right)}{(\alpha\beta-(1+\alpha)\rho)(-\beta\Gamma+(1+\alpha)(\beta-\Gamma)\rho)},\\
&M=\frac{-\alpha\beta^2\Gamma+(1+\alpha)\beta(\Gamma+\alpha(-\Gamma+\beta(2+\alpha+\Gamma)))\rho-(1+\alpha)^2(\beta+(-1+\beta)\Gamma)\rho^2}{(1+\alpha)\rho(-\beta\Gamma+(1+\alpha)(\beta-\Gamma)\rho)(\rho+\alpha(-\beta+\rho))},\\
&A=\frac{1}{\beta}-\frac{\alpha}{\rho+\alpha\rho},\\
&E=\frac{\alpha\beta^2\Gamma-(1+\alpha)\beta(\Gamma+\alpha(-\Gamma+\beta(2+\alpha+\Gamma)))\rho+(1+\alpha)^2(\beta+(-1+\beta)\Gamma)\rho^2}{\beta(\alpha\beta-(1+\alpha)\rho)(-\beta\Gamma+(1+\alpha)(\beta-\Gamma)\rho)}.
\end{aligned} \tag{7}$$

Solution 5,6: Unphysical solution and *Silenced* state

$$\begin{aligned}
&S=-\frac{\Gamma+\rho-\Gamma\rho\pm\sqrt{-4\alpha\Gamma\rho+(\Gamma(-1+\rho)+\rho)^2}}{2\Gamma\rho},\\
&M=0,\\
&A=\frac{\Gamma(-1+\rho)+\rho\pm\sqrt{-4\alpha\Gamma\rho+(\Gamma(-1+\rho)+\rho)^2}}{2\Gamma\rho},\\
&E=0.
\end{aligned} \tag{8}$$

In the presence of small basal rates ρ_0 and β_0 these solutions will be modified whereby the solutions will be mapped one-to-one to a new set but the overall structure of the solutions are not altered. The equations for the model with such basal rates are

$$\frac{dS}{dt}=(\rho_0+\rho S)\,(1-S-A-M-E)-S, \tag{9}$$

$$\frac{dA}{dt}=\alpha\,(1-S-A-M-E)-\Gamma\,S\,A-(\beta_0+\beta\,E)\,A-A, \tag{10}$$

$$\frac{dM}{dt}=(\beta_0+\beta E)\,(1-S-A-M-E)-\alpha\,M-M, \tag{11}$$

$$\frac{dE}{dt}=(\beta_0+\beta\,E)\,A+\alpha\,M-E. \tag{12}$$

We call the model **Model II**. An important observation however, is that suppression of basal rates ρ_0 and β_0 is favorable in producing a larger volume of bistable region in phase space. As an example, assigning $\beta_0=\rho_0=0.25$ in Model II, a significant reduction of the area of bistability is observed when compared to Model I for the same choice of rest of the parameters; compare Fig. 1 and Fig. 6 (c).

One can easily check that the cooperative terms proportional to ρ and Γ are essential for bistability with respect to active and silenced states. However, one can achieve bistability by setting β to zero but retaining a nonzero basal rate β_0. This model can also be analytically solved and is critical in our understanding of the engineering role played by multiple active marks and the putative cooperativity in methylation. We call this model **Model III** and the

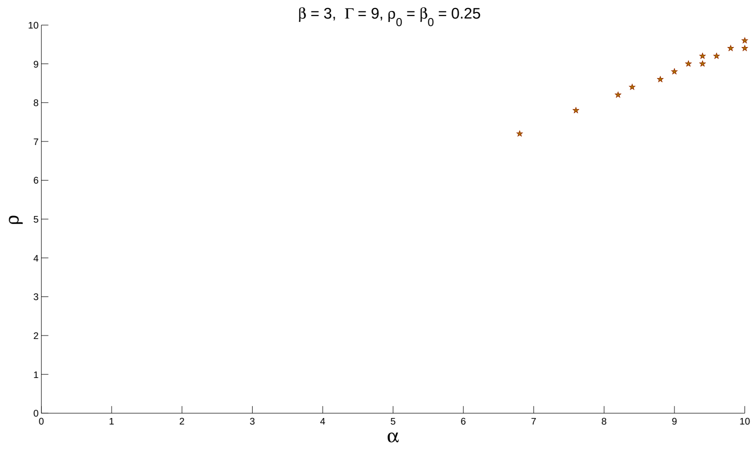

FIG. 1. Slice of phase diagram for $\alpha - \rho$ showing the bistable region for $\beta_0 = \rho_0 = 0.25$ and other parameters same as that of Fig. 6 (c). The volume of bistable region reduces on introducing basal rates ρ_0 and β_0.

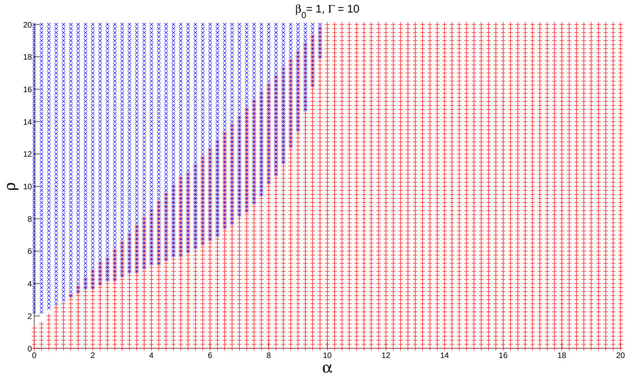

FIG. 2. Slice of phase diagram for $\beta_0 = 1$, $\beta = 0$ and $\Gamma = 10$

corresponding mean-field equations are

$$\frac{dS}{dt} = \rho\, S\,(1 - S - A - M - E) - S, \tag{13}$$

$$\frac{dA}{dt} = \alpha\,(1 - S - A - M - E) - \Gamma\, S\, A - \beta_0\, A - A, \tag{14}$$

$$\frac{dM}{dt} = \beta_0(1 - S - A - M - E) - \alpha\, M - M, \tag{15}$$

$$\frac{dE}{dt} = \beta_0\, A + \alpha\, M - E. \tag{16}$$

This set of equations can be solved exactly. In Fig. 2 a slice of the $\alpha - \rho$ phase diagram for $\beta_0 = 1$ and $\Gamma = 10$ is plotted. We do not present the exact solutions of Model III. These exact solutions were used to generate the phase diagram in Fig. 2. All the solutions have simultaneously non-zero S and E states. Model III is of limited practical interest because though the system is bistable, stochastic simulation reveals that the silenced and active regions are not well separated, see Fig. 3 where the simulation result for a choice of parameters that produce bistability is presented.

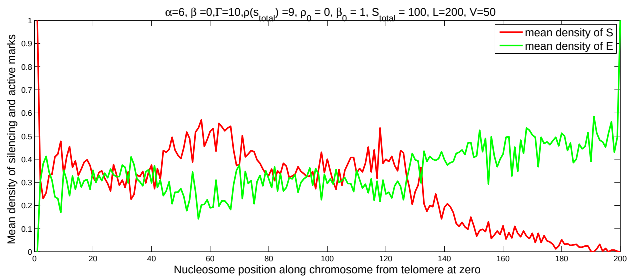

FIG. 3. Stochastic simulation showing the spatial profile of silencing and active marks for $\beta_0 = 1, \alpha = 6, \beta = 0, \Gamma = 10, \rho = 9$.

MODEL PHASE SPACE AND PERTURBATIONS

Nature of Sir mutants and inhibition of Sir activity

The parameters ρ_0, ρ and Γ describe the activity of Sir proteins. We have avoided complicating our model by separating modeling the Sir proteins, a couple of observations are in order. The parameter ρ_0 is the basal rate of Sir binding or nucleation. It is high in 'nucleation centers', for example, Sir1p recruitment by other complexes like Orc, Abf1 and Rap1 at the telomeres. Both ρ_0 and ρ are proportional to the ambient concentration of the Sir2p, Sir3p and Sir4p. The parameter ρ controls the cooperative recruitment of Sir proteins, say Sir2p, Sir3p and Sir4p by nucleosome bound Sir complex. Such cooperativity has been argued in a polymerization-like model of silencing spreading. Moreover, we have explored that such cooperativity can also arise from integrating out a detailed model where the concentration of the Sir2-4p are separately accounted for. Whatever is the microscopic mechanism of the cooperativity, the ρ term turns out to be essential for achieving a bistable region of phase space with *silenced* state as one of the stable states.

Sir3 and Sir4 mutants

For the model described by 1, typical $\alpha - \Gamma$ cross sections of phase space is presented in Fig. 4. The region of phase space where the four states are stable are represented. The wedge of multi-colored region is where the *active* state (colored green) and the *silenced* state (colored red) are both stable. Robust design principles would dictate

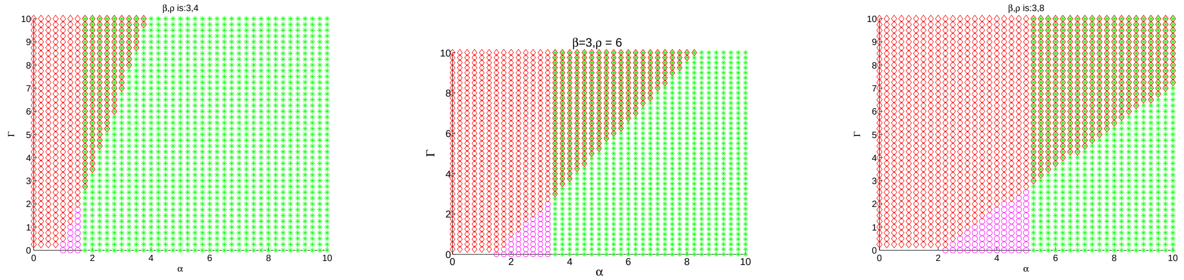

(a)$\beta = 3$ and $\rho = 4$. (b)$\beta = 3$ and $\rho = 6$. (c)$\beta = 3$ and $\rho = 8$.

FIG. 4. Slices of phase diagram in $\alpha - \Gamma$ plane. The three panels are for increasing values of ρ from left to right, with fixed β. The red diamonds is the region of stability of the *silenced* state, the green stars of the *active* state and the magenta circles the *bivalent* state.

that the wild type system remains bistable against small perturbations. Such a system corresponds to tuning the parameters (a point in phase space) such that a reasonably large volume (in phase space) of bistability around this point is ensured. We do not know what this typical choice of parameters is, however, using the phase diagram we can understand the fate of the most probable choices of parameters. For example, reducing ρ maintaining α and Γ fixed would typically push the system that was initially in a bistable region of phase space to a monostable region where only the *active* state is stable. This for example, would be the consequence of reducing the supply of the Sir proteins

involved in spreading of silencing. In the Sir3 mutant ρ is reduced drastically. Not surprisingly, the model predictions are consistent with expectations on the Sir3 mutant.

We have discussed in the main text how the limited supply of Sir proteins control ρ leading to interesting titration effects and ectopic silencing. To recapitulate the main ideas: the reaction rate ρ is proportional to the concentration of ambient Sir proteins and in telomeres where no obvious boundary elements are present to limit the spread of silencing proteins, the concentration of Sir proteins self-adjust such that the the system is tuned to the *zero velocity line*. This line in phase space are choices of parameters for which a boundary between the spread of Sir proteins and active region is stochastically balanced. Away from this line but still within a bistable region either the *active* state or the *silenced* state spread. Such a self-adjustment determines the stochastic spread and boundary of silenced state in telomeres. Denoting by S the the number of Sir proteins, and s the density of Sir proteins, the equation that determines the effective $\rho \equiv \rho_{\text{ambient}}$ is

$$\begin{aligned} S_{\text{total}} &= S_{\text{bound}} + S_{\text{ambient}} \\ \rho\left(s_{\text{ambient}}\right) &= \rho \frac{S_{\text{total}} - S_{\text{bound}}}{V}, \end{aligned} \tag{17}$$

where V is the volume of the cell nucleus. One needs to go beyond the mean-field treatment to illuminate the role of ρ self-adjusting under a limited supply of Sir proteins. We present results of a stochastic simulation to illustrate the nucleation and spreading of silencing in wild-type and the Sir3 mutants. We choose $V = 50$, $L = 200$ and $S_{\text{total}} = 100$ for these simulations. We allowed ρ to self-adjust as Sir proteins bind to the nucleosome; ρ is linear in the ambient Sir density.

In Fig. 5 (a) we plot the result of stochastic simulation. The initial value of ρ is chosen such that the system is in the bistable regions. The boundary condition is fixed to be Sir-nucleated at the left-most position and active in the rightmost position. The equilibrated profile of the mean local density $\mathcal{P}_i(S)$ and $\mathcal{P}_i(E)$ is plotted, where the mean is taken over a few hundred configurations that the system stochastically explores after equilibrating from an initial phase of spreading of silencing/active marks. A clear boundary between silenced and active region of chromosome is observed. For exactly the same choice of parameters and other configurations, but on reducing the cooperative

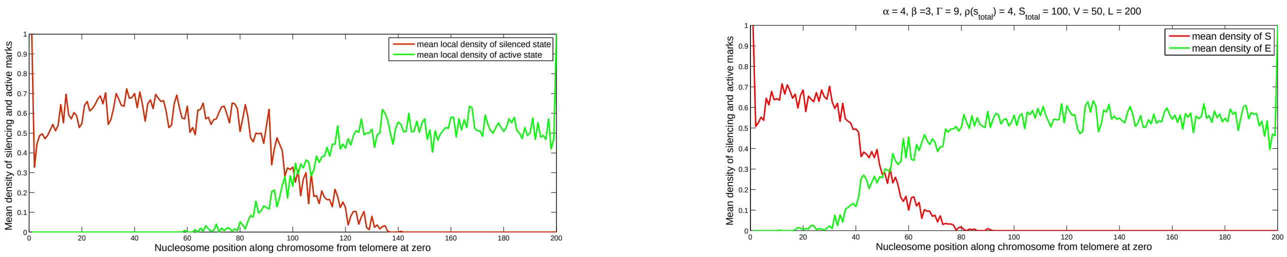

(a)$\alpha = 4, \beta = 3, \Gamma = 9, \rho(s_{\text{total}}) = 7$.

(b)$\alpha = 4, \beta = 3, \Gamma = 9, \rho(s_{\text{total}}) = 5$.

FIG. 5. Spatial profile of density of silencing and active marks as cooperative Sir binding $\rho(s_{\text{total}})$ is reduced. We chose a length of lattice (telomeric chromosome region) with 200 sites (nucleosomes) where the first site from left is fixed to be in state S, acting as the nucleation center. The right-most site is fixed to be in state E. We considered 400 samples of time evolution after an initial equilibration of the system. The left panel shows the profile for a choice of $\rho(s_{\text{total}})$ for which the system is in the bistable region, and the right panel is for reduced $\rho(s_{\text{total}})$ but the system still bistable.

recruitment rate of Sir proteins, i.e., $\rho(s_{\text{total}})$, we observe that the silencing profile is pushed back towards the telomere and most of the chromatin is active, see Fig. 5 (b). This is the effect of disrupting the cooperative recruitment of Sir proteins where ρ is reduced drastically.

Inhibition of Sir2 activity and nature of Sir2 mutants

The parameter Γ controls the deacetylation activity of Sir2 and inhibition of Sir2 deacetylation activity (for example, by Nicotinamide, Anacardic Acid or Splitomycin etc.) can tune this parameter. In Sir2 mutants, Γ is zero. Typical $\rho - \alpha$ cross-sections of the phase diagram for fixed β and three values of Γ are presented in Fig. 6 and cross-sections for fixed ρ and varying Γ are presented in Fig. 7.

A posteriori, reduction of the volume of phase space for non-zero basal rates ρ_0 and β_0 discussed in Analytical Results, justifies our considering the cooperative rates ρ and β to be the more relevant parameters and presenting

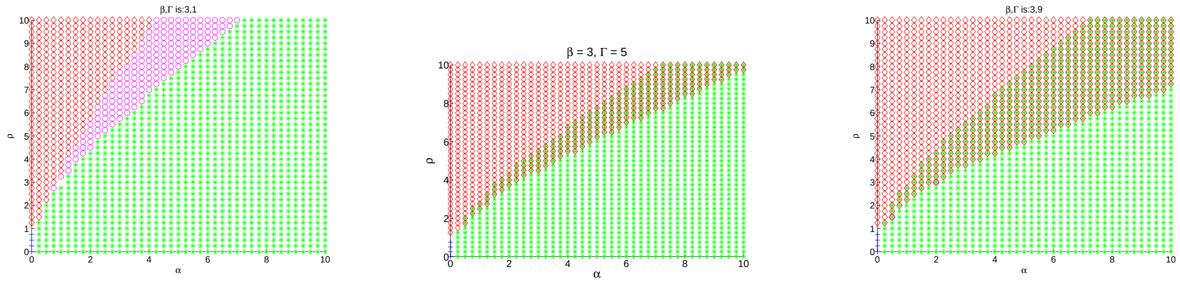

(a)$\beta = 3$ and $\Gamma = 1$. (b)$\beta = 3$ and $\Gamma = 5$. (c)$\beta = 3$ and $\Gamma = 9$.

FIG. 6. Slices of phase diagram in $\rho - \alpha$ plane. Recall that the red diamonds is the region of stability of the *silenced* state, the green stars of the *active* state, the magenta circles the *bivalent* state and the blue grid is the region of stability of the *intermediate* state. The three panels are for increasing values of Γ from left to right, with β fixed.

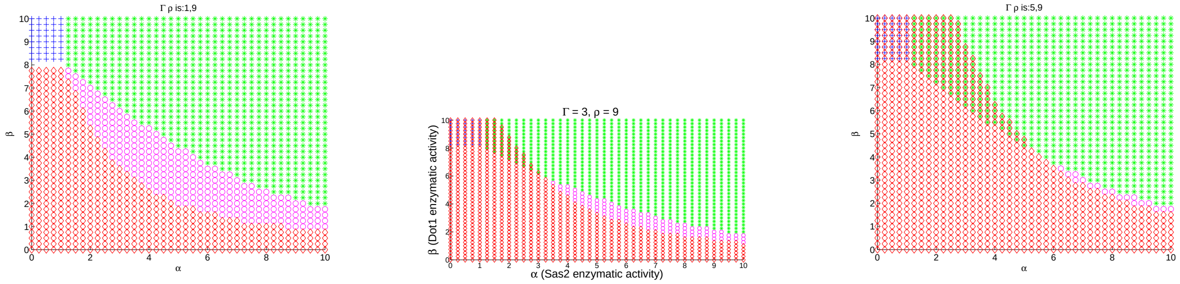

(a)$\rho = 9$ and $\Gamma = 1$. (b)$\rho = 9$ and $\Gamma = 5$. (c)$\rho = 9$ and $\Gamma = 9$.

FIG. 7. Slices of phase diagram in $\beta - \alpha$ plane. The three panels are for increasing values of Γ from left to right, and fixed ρ.

the result of the extreme case of ρ_0 and β_0 to be zero for which the model is analytically tractable. Moreover, a bigger volume of bistable region in phase space implies robustness against perturbations and noise (lesser fine-tuning of parameters). The parameter ρ_0 controls nucleation of silencing, and is tightly controlled; β_0 controls basal Dot1 driven methylation which may be low and feedback from other active marks or transcriptional activity seems to be necessary.

Stochastic simulation reveal that the front of silencing is noisier even for small values of ρ_0 and β_0. In Fig. 8 we plot the mean of densities of silencing and active marks (taken over multiple stochastic runs after initial period of spreading/equilibration of silencing). Observe that the boundary between silenced and active region is sharper for the model with putative basal rates being zero.

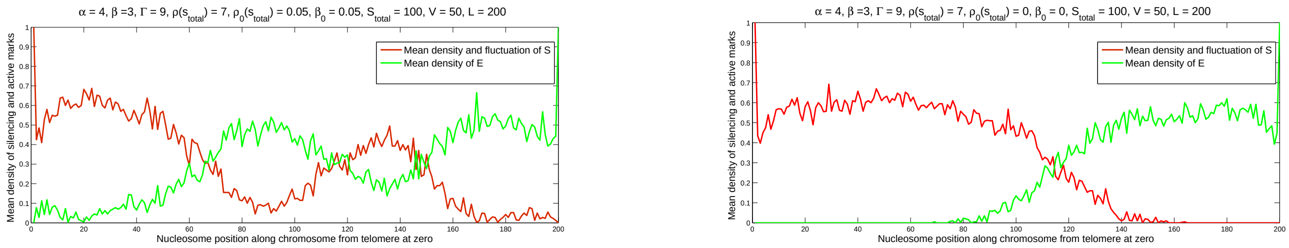

(a)$\alpha = 4, \beta = 3, \Gamma = 9, \rho(s_{\text{total}}) = 7, \rho_0(s_{\text{total}}) = 0.05, \beta_0 = 0.05$. (b)$\alpha = 4, \beta = 3, \Gamma = 9, \rho(s_{\text{total}}) = 7, \rho_0(s_{\text{total}}) = 0, \beta_0 = 0$.

FIG. 8. Comparison of density profile of silencing and active marks for non-zero and zero basal rates $\rho_0$and β_0. The left panel shows the density profiles for small basal rates β_0 and ρ_0 and right panel for zero basal rates. Small basal rates are capable of disrupting the establishment of sharp silenced and active domains.

Nature of the Sas2 mutant and inhibition of Sas2 activity

In our model, reducing the parameter α amounts to reducing Sas2 acetylase activity and Sas2 mutants this parameter should be nearly zero. Though we do not know the value of α in wild type cells, we can investigate within our model the qualitative behavior of the system for different values of α. In Fig. 9 we present typical $\rho - \beta$ cross section of the phase diagram for fixed Γ and varying values of α. Not surprisingly, a typical choice of parameters for the system that would put it initially in the bistable region for high enough α, see Fig. 9 (c) ends up in the silenced region and eventually the system loses bistability when α is reduced, see Fig. 9 (a). In order to appreciate the effect of finite

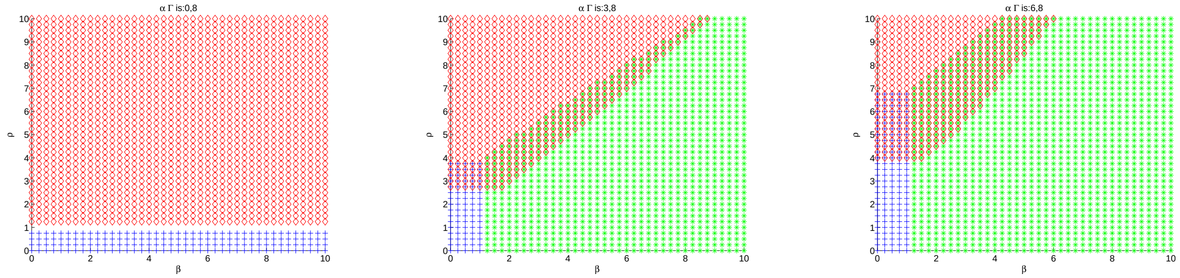

(a)$\alpha = 0$ and $\Gamma = 8$. (b)$\alpha = 3$ and $\Gamma = 8$. (c)$\alpha = 6$ and $\Gamma = 8$.

FIG. 9. Slices of phase diagram in $\rho - \beta$ plane for fixed Γ and varying α. The three panels are for increasing values of α from left to right, with fixed Γ.

supply of silencers and the titration effect, note that on reducing α there are more silencers that are nucleosome bound, which in turn reduces the the value of $\rho(s_{\text{ambient}})$. Therefore, the system dynamically equilibrates to a different value of ρ when α is changed. We determine this zero velocity line in numerical simulations. When the system parameters self-tune to the zero velocity line, the silencing front is stochastically stationary. In Fig. 10 we present the zero velocity line superimposed on the relevant cross section of the phase diagram. Stochastic simulation reveals that the on reducing α such that the system ends up in the monostable region where S state is stable, the Sir proteins are diluted out in the telomeric region and silenced region is pushed back. We explore the mean density of bound Sir proteins in stochastic simulations, and the results are shown for two different choice of parameter α, see Fig. 11. This defect is aggravated when one includes even very small value of basal rate ρ_0 of Sir protein binding (data not shown).

Nature of Dot1 mutants and the bivalent state

We present the cross sections along of the phase space with ρ and α as axis and varying values of β and intermediate Γ in Fig. 12 and for low Γ in Fig. 13.

Notice in Fig. 12 that it is only beyond a critical β that a stable *active* state emerges in the model.

In order to understand the nature of the phase space and the bifurcations, we present the unstable states across the phase space in conjunction with the stable states (that we have presented so far). For ease of comparison, we reproduce the $\beta - \rho$ cut of phase space for fixed α and Γ from the main text in Fig. 14(a), display the solutions with one unstable direction in Fig. 14(b), and two unstable directions in Fig. 14(c).

Nature of bifurcations and critical lines

In the main text we mentioned that (for Model I) the active state is stable only for $\beta > \beta_{\text{critical}}$. The derivation is as follows. As is clear from Fig. 14(a), the *intermediate* state exchanges stability with the *active* state at β_{critical}. It

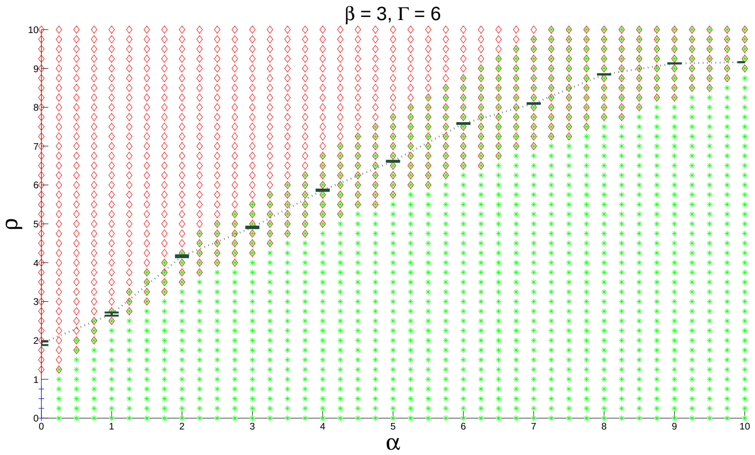

FIG. 10. Slice of phase diagram for $\alpha - \rho$ showing the bistable region for $\beta = 3$ and $\Gamma = 6$. For limited supply of silencer proteins the system self tunes the parameter $\rho(s_{\text{ambient}})$ for changing α to settle on the zero velocity line drawn in dotted back line.

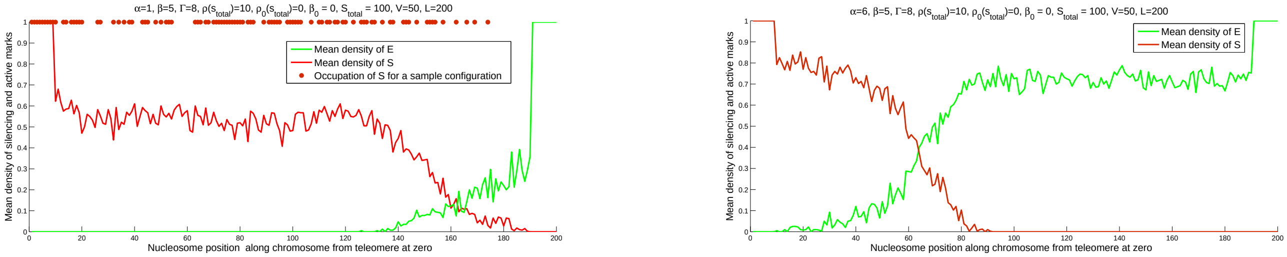

(a)$\alpha = 0.5, \beta = 5, \Gamma = 8, \rho(s_{\text{total}}) = 10.$ (b)$\alpha = 6, \beta = 5, \Gamma = 8, \rho(s_{\text{total}}) = 10.$

FIG. 11. Comparison of profile of silencing marks for Sas2 mutant and wild type. Silencing is reduced in Sas2 mutant. Th eleft panel is for very small α mimicing Sas2 mutants, the right panel is for "wild-type" values of α, i.e., the system's parameters are deep in the bistable region.

is easy to check that the eigenvalues of the Jacobian matrix determining stability of the *intermediate* state are,

$$\Big[-(1+\alpha), \tag{18}$$

$$\frac{-2-\alpha^2+\alpha(\beta-3) \pm \sqrt{\alpha\left(\alpha^3+4\beta+2\alpha^2+2\alpha^2\beta+\alpha+6\alpha\beta+\alpha\beta^2\right)}}{2(1+\alpha)}, \tag{19}$$

$$\left(-1+\frac{\rho}{1+\alpha}\right)\Big]. \tag{20}$$

Clearly, for $\rho < (1+\alpha)$ the intermediate state is stable, and is unstable otherwise. It can be shown, and is clear from Fig. 14(a) that the silenced state is stable for $\rho > (1+\alpha)$. Similarly, the other eigenvalue can be used to obtain $\beta_{\text{critical}} = \frac{(1+\alpha)^2}{\alpha(2+\alpha)}$.

We are mainly interested in the bivalent state and the bifurcations related to it. In order to understand the bifurcations associated with *active*, *silenced* and *bivalent* states, we plot the density of S marks, $P(S)$ fixing α, Γ and ρ and for varying β. The cuts are for two fixed values of ρ in Fig. 14(a):

1. For high ρ such that for increasing β the systems transitions from silenced-stable to bivalent-stable to active stable, in Fig. 15(a).

2. For low ρ such that for increasing β the system transitions from silenced-stable to bistable to active-stable regions, in Fig. 15(b).

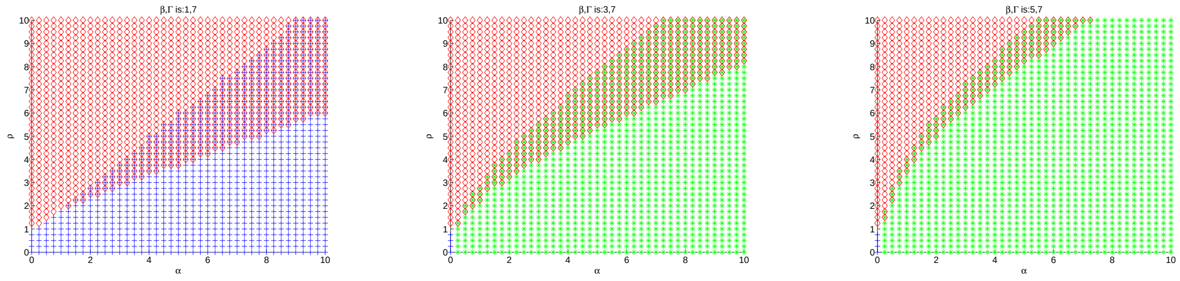

(a)$\beta = 1$ and $\Gamma = 7$. (b)$\beta = 3$ and $\Gamma = 7$. (c)$\beta = 5$ and $\Gamma = 7$.

FIG. 12. Slices of phase diagram in $\rho - \alpha$ plane for intermediate Γ. The three panels are for increasing values of β from left to right, and fixed Γ. Recall that the red diamonds represent the region of stability of the *silenced* state, the green stars of the *active* state, the magenta circles the *bivalent* state and the blue grid is the region of stability of the *intermediate* state.

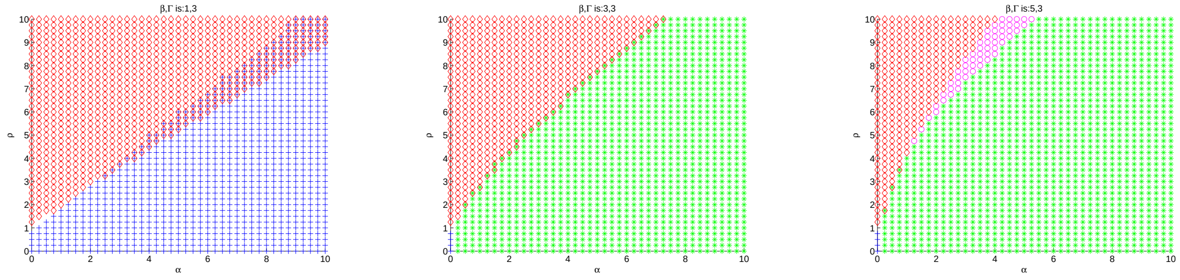

(a)$\beta = 1$ and $\Gamma = 3$. (b)$\beta = 3$ and $\Gamma = 3$. (c)$\beta = 5$ and $\Gamma = 3$.

FIG. 13. Slices of phase diagram in $\rho - \alpha$ plane for low Γ. The three panels are for increasing values of β from left to right, and fixed Γ.

The bifurcations are *transcritical*, whereby the *bivalent* state exchanges stability with the active and silenced state. We have only shown the physical solutions (probabilities $\mathcal{P}(S), \mathcal{P}(A), \mathcal{P}(M)$, and $\mathcal{P}(E)$ real and sum up to one) in the Fig. 15(b) and Fig. 15(a).

The transcritical bifurcation will become a crossover in the presence of non-zero basal rates ρ_0 and β_0. However, owing to the reduced volume of bistable region in the phase space for moderate non-zero basal rates, as discussed above, we think that the physical system maintains low basal rates. Therefore, the character of the *bivalent* state remains relevant and mostly unchanged though the nature of the bifurcation changes.

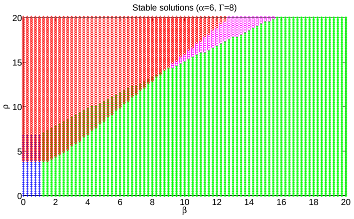

(a)Slice of phase diagram for $\beta - \rho$ showing all stable states. Red diamonds represent region where *silenced* state, green stars where *active* state, magenta circles where *bivalent* state, blue crosses where *intermediate* state are stable. Regions of overlap of symbols are bistable regions.

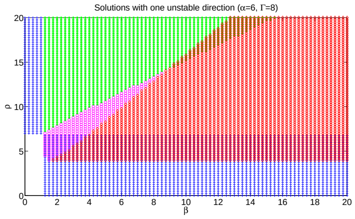

(b)Slice of phase diagram for $\beta - \rho$ showing all states unstable in only one direction. Red diamonds represent region where *silenced* state, green stars where *active* state, magenta circles where *bivalent* state, blue crosses where *intermediate* state are unstable in only one direction.

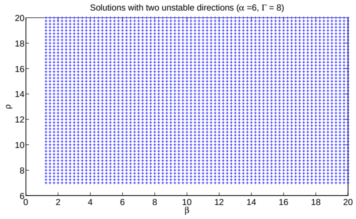

(c)Slice of phase diagram for $\beta - \rho$ showing all states unstable in only two directions. Blue crosses are regions where *intermediate* state is unstable in two direction. It is the only state with two unstable directions.

FIG. 14. Showing all solutions in phase space, stable and unstable.

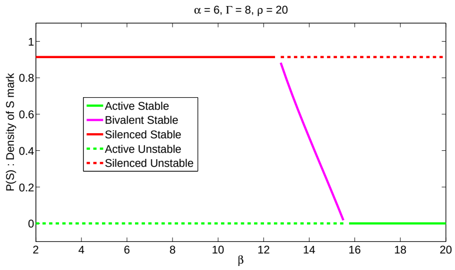

(a)Cut for $\rho = 20$ in Fig. 14(a) showing density of silencing marks transitioning between silenced, bivalent and active marks.

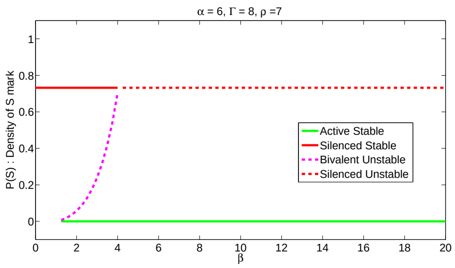

(b)Cut for $\rho = 7$ in Fig. 14(a) showing density of silencing marks transitioning between silenced, bivalent and active marks.

FIG. 15. Cuts for fixed ρ, α and Γ for varying β, showing the density of S marks at and near the transcritical bifurcation.
